# Intensity modulated proton therapy for early-stage glottic cancer: high-precision approach to laryngeal function preservation with exceptional treatment tolerability

**DOI:** 10.1186/s13014-022-02144-w

**Published:** 2022-12-05

**Authors:** Thomas Held, Henrik Franke, Kristin Lang, Tanja Eichkorn, Sebastian Regnery, Katharina Weusthof, Lukas Bauer, Karim Plath, Gerhard Dyckhoff, Peter K. Plinkert, Semi B. Harrabi, Klaus Herfarth, Jürgen Debus, Sebastian Adeberg

**Affiliations:** 1 Department of Radiation Oncology, Heidelberg University Hospital, Im Neuenheimer Feld 400, 69120 Heidelberg, Germany; 2 Heidelberg Institute of Radiation Oncology (HIRO), Heidelberg, Germany; 3 National Center for Tumor diseases (NCT), Heidelberg, Germany; 4 Clinical Cooperation Unit Radiation Oncology, German Cancer Research Center (DKFZ), Heidelberg, Germany; 5 Heidelberg Ion Beam Therapy Center (HIT), Heidelberg, Germany; 6 German Cancer Consortium (DKTK), German Cancer Research Center (DKFZ), Heidelberg, Germany; 7 Department of Otorhinolaryngology, Head and Neck Surgery, University of Heidelberg, Heidelberg, Germany

**Keywords:** Head and neck cancer, Laryngeal cancer, Squamous cell carcinoma, Proton therapy, Glottic cancer, Radiotherapy, Treatment toxicity, Laryngeal preservation

## Abstract

**Background:**

Due to the increasing expertise in transoral laser surgery and image-guided radiation therapy, treatment outcomes have recently improved in patients with early-stage glottic cancer. The objective of the current study was to evaluate intensity-modulated proton therapy (IMPT) as novel treatment option.

**Methods:**

A total of 15 patients with T1-2N0 glottic squamous cell carcinoma, treated between 2017 and 2020, were evaluated. Toxicity was recorded according to the Common Terminology Criteria for Adverse Events (CTCAE) v4.03.

**Results:**

The majority were T1a/b tumors (66.7%) and no patient had lymph node or distant metastases. The median total dose was 70 Gy relative biological effectiveness (RBE) (range 66–70 Gy RBE). The one- and two-year OS and metastases-free survival were 100%. One patient developed local failure and received salvage laryngectomy. No higher-grade acute or late toxicity was reported. The mean number of CTCAE grade I and II overall toxicity events per patient was 4.1 (95%-[confidence interval] CI 3.1–5.3) and 1.0 (95%-CI 0.5–1.5).

**Conclusion:**

High-precision proton therapy of T1-2N0 glottic cancer resulted in exceptional treatment tolerability with high rates of laryngeal function preservation and promising oncological outcome. IMPT has the potential to become a standard treatment option for patients with early-stage laryngeal cancer.

## Background

The larynx is one of the most highly innervated organs in humans and serves multiple substantial, complex and highly evolved biological functions [1]. These include among others nutrition, airway protection, communication and expression of emotions. Laryngeal cancer affects an estimated 177,000 patients per year worldwide and leads to around 94,000 attributable deaths annually [2]. In patients with early (stage I and II) laryngeal cancer, glottic cancers have the most favorable outcome with 5-year overall survival (OS) rates between 80 and 95% [3], emphasizing function preservation in these patients. With the declining use of open surgery [4], transoral laser microsurgery (TLM) and intensity modulated radiation therapy (IMRT) have recently improved functional preservation. In general, the oncological and functional outcome in patients with early-stage glottic cancer are comparable between TLM and radiotherapy (RT) [5]. Several factors, among others laryngeal function, tumor site, patient age and comorbidities, clinical expertise, rehabilitation resources, and patient preference are currently influencing the initial clinical management. Patients who receive surgery may require postoperative RT due to positive margins. While initial TLM preserves the option of RT in the case of local recurrence, salvage-surgery in a previously irradiated field may have higher risks of complications. Despite several clinical studies, data on TLM versus RT in early-stage glottic cancer remain controversial [6–8]. Unequivocally, the main factor determining the post-treatment functional outcome is the best possible sparing of healthy tissue uninvolved by the tumor. Current developments include high-precision RT techniques to minimize the dose to the (contralateral) normal tissue and adjacent organs at risk, such as the arytenoid cartilage or carotid arteries [9]. Since functional outcome is influenced by the extent of the irradiated volume, intensity-modulated proton therapy (IMPT), due to its biophysical advantages compared to IMRT, may further reduce toxicity and improve laryngeal preservation [10]. The objective of the current study was to evaluate the outcome, acute and late toxicity, as well as the rate of laryngeal function preservation in early-stage glottic carcinoma treated with IMPT.

## Methods

### Screening and patient selection

A total of 7300 patients treated with particle therapy at our institution were screened, after approval by the regional ethics committee. Patients with early-stage localized glottic cancer were selected for IMPT at the discretion of the treating radiation oncologist team. All patients with T1-2N0 glottic squamous cell carcinoma treated with proton therapy between 2017 and 2020 were included in this retrospective analysis. Patients with other tumor entities, e.g. chondrosarcoma or adenoid cystic carcinoma, were excluded from the current study. A total of 15 patients were evaluated.

### Treatment planning

All patients were immobilized using a thermoplastic head mask system. Computed tomography (CT) scans with 3-mm or 1-mm slice thickness were used for treatment planning. Contrast-enhanced CT or T1-weighted magnetic resonance imaging (MRI) was used for target volume delineation. Treatment planning was conducted using Syngo PT Planning version 10 (Siemens, Erlangen, Germany) or RayStation (RaySearch Laboratories, Stockholm, Sweden).

The gross tumor volume (GTV) and clinical target volume (CTV) were defined according to the established International Commission on Radiation Units and Measurements (ICRU) [11]. The target volume delineation was conducted in adherence to existing clinical consensus guidelines for laryngeal cancer [12]. The CTV1 included the GTV with a margin of 5 mm. The CTV2 included the CTV1 with a margin of 5 mm and the glottic and/or sub-/supraglottic larynx, depending on the localization. A margin of 3 mm was added for the planning target volume (PTV). Dose prescription was conducted according to current clinical guidelines [13] and dose constraints of normal tissues were respected according to the Quantitative Analyses of Normal Tissue Effects in the Clinic [14].

All patients were treated using a rotational gantry beam delivery system and the beam setup was individualized for each patient to optimize robustness and reduce the dose surrounding organs at risk. For protons, a fixed relative biological effectiveness (RBE) of 1.1 was used for dose calculation, according to current guidelines [15]. Daily orthogonal X-rays were used for daily image-guidance prior to irradiation followed by position correction. In addition, weekly CT-scans were performed to further evaluate anatomical and positional changes. At the discretion of the treating radiation oncologist, replanning was performed to improve target volume coverage and/or normal tissue sparing. Highly conformal, IMPT was enabled using the beam scanning method with active energy variation for dose-depth distribution.

### Follow-up

Patients received follow-up imaging with contrast-enhanced MRI or CT of the head and neck six to eight weeks after treatment and then every three months within the first two years post-treatment. To rule out metastases, a CT thorax scan and abdominal sonography was performed once per year. Symptoms and toxicity were recorded non-standardized by a radiation oncologist according to the Common Terminology Criteria for Adverse Events (CTCAE) version 4.03. In addition, the patients presented to the ear, nose, and throat (ENT) specialist for clinical examination for every follow-up.

### Statistical analysis

Statistical analysis was performed using R version 4.0.3 (www.r-project.org). Clinical data was derived from the Heidelberg Ion Beam Therapy Center (HIT) cancer database. Patient and treatment characteristics were evaluated using descriptive statistics. In addition to conventional toxicity analysis according to CTCAE v4.03, the mean numbers of toxicity events per patient were evaluated based on the TAME method [16]. The median follow-up for overall survival (OS) was calculated using the inverse Kaplan-Meier method. The OS and local and distant progression-free survival (L-PFS, D-PFS) were evaluated from the end of IMPT to the occurrence of tumor progression detected via imaging and/or clinical examination using the Kaplan-Meier method. Prognostic factors for OS and L-PFS were determined using the log-rank test. Multivariate analysis was conducted with the Cox-regression model and a *p*-value < 0.05 was considered statistically significant. The laryngo-esophageal dysfunction-free survival (LE-DFS) was evaluated from the end of IMPT and included the events death, local relapse, total or partial laryngectomy, tracheotomy or feeding tube placement [17].

## Results

### Patient and treatment characteristics

All patients in the current study had glottic squamous cell carcinomas. The majority were T1a/b tumors (66.7%) and no patient had affected lymph node or distant metastases. A total of nine patients (60.0%) were current smokers with a median of 40 reported pack-years (range 7–150 pack-years). Most patients had a good performance status (median Karnofsky Performance Score [KPS] 90%) prior to IMPT. No patient received a gastric feeding tube or tracheostomy during or after IMPT. All patients received logopedic counseling or treatment during and after IMPT. The median time interval from initial diagnosis to IMPT was 2.4 months (range 0.6–78.4 months). A total of 7 patients (47%) received definitive RT due to residual or recurrent cancer after surgical resection. Detailed patient and treatment characteristics are summarized in Table 1.

**Table 1:** Patient and treatment characteristics (n = 15 patients)

	Patients	%
*Sex*		
Female	1	6.7
Male	14	93.3
T-stage prior to RT		
T1a	6	40.0
T1b	4	26.7
T2	5	33.3
*Smoking*		
≥ 10 pack-years	8	53.3
< 10 pack-years	1	6.7
Never	6	40.0
*RT setting*		
Additive	2	13.3
Definitive	13	86.7
*Gantry beam angle*		
30°/330°	1	6.7
40°/320°	6	40.0
50°/310°	7	46.6
60°/320°	1	6.7

	Median	Range
Age prior to RT (years)	68	35–83
KPS prior to RT (%)	90	60–100
BMI prior to RT (kg/m²)	25.9	18.4–30.4
BMI post RT (kg/m²)	24.9	18.1–30.4
Total dose (Gy RBE)	70	66–70
Single dose (Gy RBE)	2.0	1.8–2.3
RT fractions	35	30–35
Treatment time per fraction (min)	6	4–9
PTV (ccm)	63.2	16.0–183.9
CTV (ccm)	22.9	3.4–128.4
GTV (ccm)	1.8	0.4–7.4
Total number of scan spots	4306	1236–11641
Maximum energy protons (MeV/u)	109	91–127

*RT* Radiotherapy, * IMPT* Intensity modulated proton therapy,* KPS* Karnofsky performance score, BMI Body mass index,* RBE* Relative biological effectiveness, *ccm* cubic centimeters, *PTV* Planning target volume, *CTV* Clinical target volume, *GTV* Gross tumor volume

**Fig. 1:**
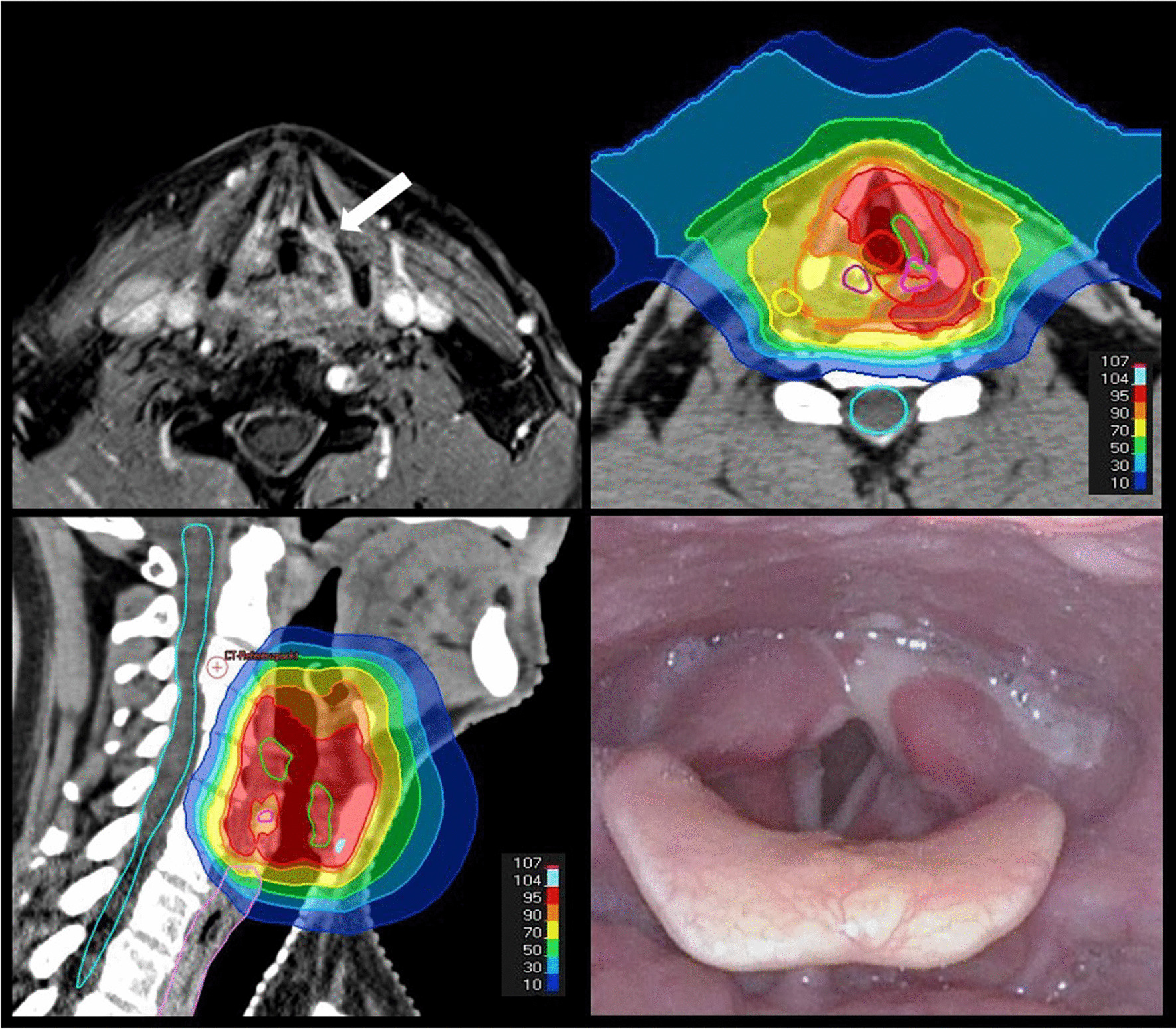
63-year-old male patient with T2N0 glottic carcinoma originating in the left vocal cord with restricted vocal cord mobility and field cancerization with severe squamous epithelial dysplasia affecting also the left supraglottis. The patient was treated with intensity modulated proton therapy (IMPT) with a dose of 63 Gy relative biological effectiveness (RBE) in 35 fractions of 1.8 Gy RBE with a simultaneous integrated boost to the tumor region up to a total dose of 70 Gy RBE with contralateral arytenoid and carotid artery sparing. In the first follow-up six weeks after IMPT, a complete remission was shown clinically and by imaging. Laryngoscopy showed laryngeal edema with fibrin coatings in the arytenoid region, the piriform sinus and the vocal cords on both sides. Although the glottic closure was still incomplete during phonation, the mobility of the vocal cords was improved

A total of 13 patients (86.7%) received definitive IMPT and two patients (13.3%) additive IMPT after R1 resection. The median total dose was 70 Gy RBE (range 66–70 Gy RBE) and 9/15 patients (60.00%) were treated with a simultaneous integrated boost concept. The optimization strategy was single beam optimization in three patients (20.0%) and IMPT in 12 patients (80.0%). One patient received 9/30 fractions using intensity modulated radiotherapy and the remaining fractions with IMPT, due to planned machine maintenance. Since the glottic larynx possesses minimal lymphatic drainage and nodal involvement is very rare, elective nodal irradiation was not performed in any patient. None of the patients received simultaneous chemotherapy. The median treatment time per fraction was 6 min (range 4–9 min). All patients were treated using a rotational gantry system with two beams. Four different beam arrangements were used, depending on the patient position and anatomy. All patients completed IMPT without interruptions or discontinuations. A clinical case of a patient with T2N0M0 glottic cancer is shown in Fig. 1.

### Local control and survival analysis

The median follow-up interval for OS after IMPT was 15.0 months (range 6.2–43.1 months). The one- and two-year OS and D-PFS were 100%. Only one patient developed local failure in-field 18 months after IMPT and received salvage laryngectomy. The one- and two-year L-PFS and LE-DFS were 92.0% and 90.0%, respectively. None of the patients developed regional or distant failure during the follow-up interval.

### Acute and late toxicity

IMPT was tolerated very well by all patients with no reported higher-grade (grade III–IV) acute or late toxicities. The most frequent grade I–II acute toxicities were dysphagia (60.0% grade I; 13.3% grade II), hoarseness (67.7% grade I; 6.7% grade II), radiation dermatitis (40% grade I; 26.7% grade II) and odynophagia (33.3% grade I; 26.7% grade II). The mean number of grade I and II acute toxicity events per patient were 3.3 (95%-[confidence interval] CI 2.5–4.2) and 0.9 (95%-CI 0.4–1.3), respectively.

The most commonly reported late toxicities were hoarseness (grade I 40.0%; grade II 6.7%) and laryngopharyngeal dysesthesia (grade I 33.3%; grade II 6.7%). The mean number of grade I and II late toxicity events per patient were 1.7 (95%-CI 1.0–2.6) and 0.1 (95%-CI 0–0.3), respectively. The mean number of grade I–II overall toxicity per patient was 5.1 (95%-CI 4.2–6.3). One patient developed acute laryngitis 29 months after IMPT and recovered quickly after treatment with i.v. antibiotics. Since the patient had multiple comorbidities and risk factors, among others continued smoking (cumulative 150 pack-years) and previous extended gastrectomy for adenocarcinoma of the gastroesophageal junction with severe reflux disease, a causal relationship with IMPT was rated as unlikely. Details on the mean number and total counts of acute, late and overall toxicity events are summarized in Tables 2 and 3.

**Table 2:** Mean number of CTCAE v4.03 toxicity events and overall toxicity per patient (n = 15)

	Mean	95%-CI	Patients	%
*Acute short-term toxicities *				
CTC grade I	3.3	2.5–4.2	6	40.0
CTC grade II	0.9	0.4–1.3	9	60.0
CTC grade I-II	4.2	3.4–5.0	15	100.0
*Late toxicities *				
CTC grade I	1.7	1.0–2.6	11	73.3
CTC grade II	0.1	0–0.3	2	13.3
CTC grade I-II	1.8	1.2–2.8	13	86.6
*Overall toxicities *				
CTC grade I	4.1	3.1–5.3	5	33.3
CTC grade II	1.0	0.5–1.5	10	66.7
CTC grade I-II	5.1	4.2–6.3	15	100.0

*CTCAE* Common Terminology Criteria of Adverse Events,* CI* Confidence interval; Comment: Acute toxicity that continued in the late phase was counted as both acute and late toxicity, but only counted once in the overall toxicity events per patient category. If a patient had more than one toxicity event, only the highest toxicity grade was counted once in the total count category

**Table 3:** Number of patients with CTCAE v4.03 grade I–II acute, late and overall toxicity (n = 15)

	Acute toxicity (%)	Late toxicity (%)	Overall (%)
Laryngopharyngeal dysesthesia	3 (20.0)	6 (40.0)	6 (40.0)
Dysphagia	11 (73.3)	3 (20.0)	12 (80.0)
Odynophagia	9 (60.0)	3 (20.0)	10 (66.7)
Pharyngeal mucositis	3 (20.0)	0 (0)	3 (20.0)
Nausea	1 (6.7)	0 (0)	1 (6.7)
Lymph edema	0 (0)	1 (6.7)	1 (6.7)
Laryngeal edema	1 (6.7)	1 (6.7)	2 (13.3)
Radiation dermatitis	10 (67.7)	0 (0)	10 (67.7)
Laryngeal hemorrhage	1 (6.7)	0 (0)	1 (6.7)
Dehydration	1 (6.7)	0 (0)	1 (6.7)
Weight loss	4 (26.7)	1 (6.7)	5 (33.3)
Cough	5 (33.3)	0 (0)	5 (33.3)
Hoarseness	11 (73.3)	7 (46.7)	12 (80.0)
Fatigue	0 (0)	3 (20.0)	3 (20.0)

*CTCAE* Common terminology criteria of adverse events,* CI* Confidence interval; Comment: Acute toxicity that continued in the late phase was counted as both acute and late toxicity, but only counted once in the overall toxicity events per patient category. If a patient had more than one toxicity event, only the highest toxicity grade was counted once in the total count category

## Discussion

The current study reports first clinical results of high-precision proton therapy in patients with early-stage glottic squamous cell carcinoma. IMPT resulted in exceptional overall treatment tolerability with high laryngeal function preservation rates and promising oncological outcome.

The two-year L-PFS and OS were 90.0% and 100%, comparable to previously reported treatment outcomes after MLS or RT in patients with early-stage glottic cancer [3]. Since none of the patients in our study required a gastric feeding tube or tracheostomy, the two-year LE-DFS, an essential outcome parameter for patients with laryngeal cancer [18], was also 90.0%. Further follow-up is necessary to confirm the long-term functional outcome and larynx preservation. Previous studies reported comparable oncological and functional outcome after MLS versus RT in early-stage glottic cancer [5, 6]. In a meta-analysis by Guimarães et al. [8], endoscopic resection showed a tendency to lower overall risk for laryngectomy while definitive RT was favorable with regard to long-term vocal quality in early-stage glottic cancer. However, most of these clinical trials did not reflect current high-precision image-guided RT and dose fractionation regimens, which yield decisive advantages with regard to toxicity [19] and dose escalation [20], compared to 3D-conformal RT. In addition, in patients with non-superficial T1-2N0 glottic cancer or diagnosed field cancerization, requiring more extensive larynx-preserving resection, definitive RT is generally preferred [3].

Previous studies could also demonstrate favorable outcomes with altered fractionation RT. In a prospective study on 180 patients with T1N0 glottic cancer [21], moderate hypofractionation compared to normofractionated RT resulted in a superior 5-year local control rate of 92% versus 77% (*p* = 0.004). Moderate acceleration (6 versus 5 weekly fractions) can also significantly improve locoregional control in patients with glottic squamous cell carcinoma [22]. Due to its biophysical features, IMPT is well suited for both moderately hypofractionated and accelerated treatment regimens, as performed in a subgroup of patients in the current study. The treatment regimens were rather heterogeneous in the current study, since uncertainties regarding the optimal total dose and fractionation remain. In the reported studies on altered fractionation, both acute and long-term toxicity rates were not significantly changed compared to normofractionation [23]. However, different approaches to toxicity reporting impede the comparison of results. Several studies evaluated only the total toxicity count per patient, e.g. the highest toxicity grade, or only reported high-grade (CTC grade III–IV) toxicities in general. Our results are based on the TAME toxicity reporting system [16], accounting for every grade and event per patient. Nevertheless, the mean number of overall acute and late toxicity events of 5.3 was encouraging and either comparable or favorable to previous studies using IMRT techniques [23]. The most important conclusion regarding toxicity in the current study is also the most obvious—no severe acute or late grade III–IV toxicities were reported. In addition, all patients completed IMPT without interruptions, which is essential for the treatment outcome in patients with glottic cancer [24].

High-precision RT of glottic cancer, e.g. IMPT, yields pronounced dosimetric advantages, in particular for the treatment of head and neck cancer [10]. Several dosimetric comparison studies for laryngeal cancer could verify the advantages of protons [25, 26], e.g. with regard to the carotid arteries, the arytenoid cartilage, the swallowing muscles, the thyroid gland, and the spinal cord. However, a major challenge of IMPT, which is particularly important for glottic cancer, is the high susceptibility to density changes due to anatomical or positional changes during RT. Thus, the increased precision of proton therapy of glottic cancer requires measurements to account for the risk of marginal misses and motion interplay effects. Therefore, prospective clinical trials including on-site image guidance are indispensable for the implementation of IMPT for the treatment of glottic cancer.

In particular in partial laryngeal irradiation of early-stage lateralized glottic cancer, image guidance with daily laryngeal soft tissue matching can increase local control considerably [20]. In addition, the dose to the contralateral vocal cord can be significantly reduced using image-guided IMRT compared to conventional 3D-conformal RT [27], resulting in a distinct decrease of treatment toxicity [19]. The potential clinical benefits of unilateral vocal cord versus complete laryngeal RT are currently evaluated as part of a prospective randomized clinical trial [9]. High-precision IMPT has the potential to improve treatment tolerability in selected patients with early-stage lateralized tumors even further. However, state-of-the-art on-site image-guidance with CT or MRI, which is increasingly availably in particle therapy centers, is required for daily soft-tissue matching, in particular for treatment regimens with altered fractionation. In addition, further radiation therapy innovations with the potential to minimize treatment toxicity while enhancing tumor control in early-stage glottic cancer, e.g. ultra-high dose rate RT [28, 29] or helium ion RT [30–32], are currently under development.

### Limitations

There are among others the following limitations of this study. First, due to the retrospective nature, the reported toxicity was recorded non-standardized and could therefore be underestimated. Second, the median follow-up interval of 17.0 months was rather short, which creates uncertainties with regard to the results of oncological and functional outcome and long-term toxicity. Third, patients often continued follow-up with their external ENT-specialist in a non-standardized setting. Nonetheless, the current study increases the body of evidence with regard to particle therapy for early-stage laryngeal cancer with direct implications for ongoing and future clinical studies.

## Conclusion

High-precision proton therapy of T1-2N0 glottic cancer resulted in exceptional treatment tolerability with high rates of laryngeal function preservation and promising oncological outcome. IMPT could become a standard treatment option for patients with early-stage laryngeal cancer who are not candidates for MLS. Further follow-up, dosimetric comparison studies and prospective randomized clinical trials with state-of-the-art image-guidance and standardized functional assessments are required to confirm these findings.

## Data Availability

The data that support the findings of this study are available from the corresponding author on reasonable request.

## Abbreviations

CI: Confidence interval
CT: Computed tomography
CTCAE: Common terminology criteria for adverse events
CTV: Clinical target volume
D-PFS: Distant progression-free survival
ENT: Ear, nose and throat
GTV: Gross tumor volume
HIT: Heidelberg Ion Beam Therapy Center
ICRU: International Commission on Radiation Units and Measurements
IMPT: Intensity-modulated proton therapy
IMRT: Intensity-modulated radiation therapy
KPS: Karnofsky performance score
LE-DFS: Laryngo-esophageal dysfunction-free survival
L-PFS: Local progression-free survival
MRI: Magnetic resonance imaging
OS: Overall survival
PTV: Planning target volume
RBE: Relative biological effectiveness
RT: Radiotherapy
TLM: Transoral laser microsurgery
